# The complete chloroplast genome of *Illicium simonsii* Maxim. (Illiciaceae), a species with important medicinal properties

**DOI:** 10.1080/23802359.2024.2356753

**Published:** 2024-05-22

**Authors:** Fuqin Zhou, Yunqi Liu, Shuang Xiong, Yuan Huang

**Affiliations:** School of Life Sciences, Yunnan Normal University, Kunming, P. R. China

**Keywords:** Chloroplast genome, *Illicium simonsii*, phylogenomic analysis

## Abstract

*Illicium simonsii* Maxim (1888) is a medicinal species of the genus *Illicium* in the Illiciaceae family. It is commonly used to cure gastro-frigid vomiting, cystic hernia, gas pains in the chest, and scabies as folk medicine. To utilize its resources efficiently, the complete chloroplast genome of *I. simonsii* was sequenced, assembled, and annotated by using high-throughput sequencing data. The complete chloroplast genome was 143,038 bp in length, with a large single-copy region (LSC) of 101,094 bp, a short single-copy region (SSC) of 20,070 bp, and a pair of inverted repeats (IRs) of 21,874 bp. A total of 113 genes were annotated, including 79 protein-coding genes, 30 tRNA genes, and four rRNA genes. The phylogenetic tree exhibited that *I. simonsii and Illicium burmanicum* form a sister group, and were nested in the monophyletic clade of the *Illicium* genus.

## Introduction

*Illicium simonsii* Maxim (1888) is an evergreen tree of the genus *Illicium* in Illiciaceae family, which is widely distributed in southwest China, northern Burma, and northeast India (Xia et al. 2008; Zhang et al. 2018). Most studies of *I. simonsii* have focused on phytochemistry, and revealed that it has significant value (Lin et al. 2007; Wu and Lin 2008; Liu et al. 2012; Guo et al. 2021). It is a poisonous plant because ansisatin (a toxic substance that causes convulsions, respiratory paralysis and death) was extracted from its leaves, flowers and fruit (Liu et al. 2012; Yang et al. 1991). However, it is a rich source of essential oils, sesquiterpenoids, lignans, and C_6_–C_3_ compounds, and has been used in traditional Chinese medicine for the treatment of rheumatic arthritis and to relieve pain (Li et al. 2022; Li et al. 2020; Zhang et al. 2019). In reality, it is difficult to reliably identify *I. simonsii* using morphologies due to the high similarity in morphological characteristics of *Illicium* species. This greatly impedes the effective development and usage of the resource of *I. simonsii*. Fortunately, the chloroplast genome has the potential to be an important source of genetic markers for species identification and evolution. (Lee et al. 2019). Therefore, it is urgent and necessary to assemble and sequence the complete chloroplast genome of *I. simonsii*, which may be crucial in solving the problem of accurate species identification and phylogenetic research.

## Materials and methods

**Figure 1.**
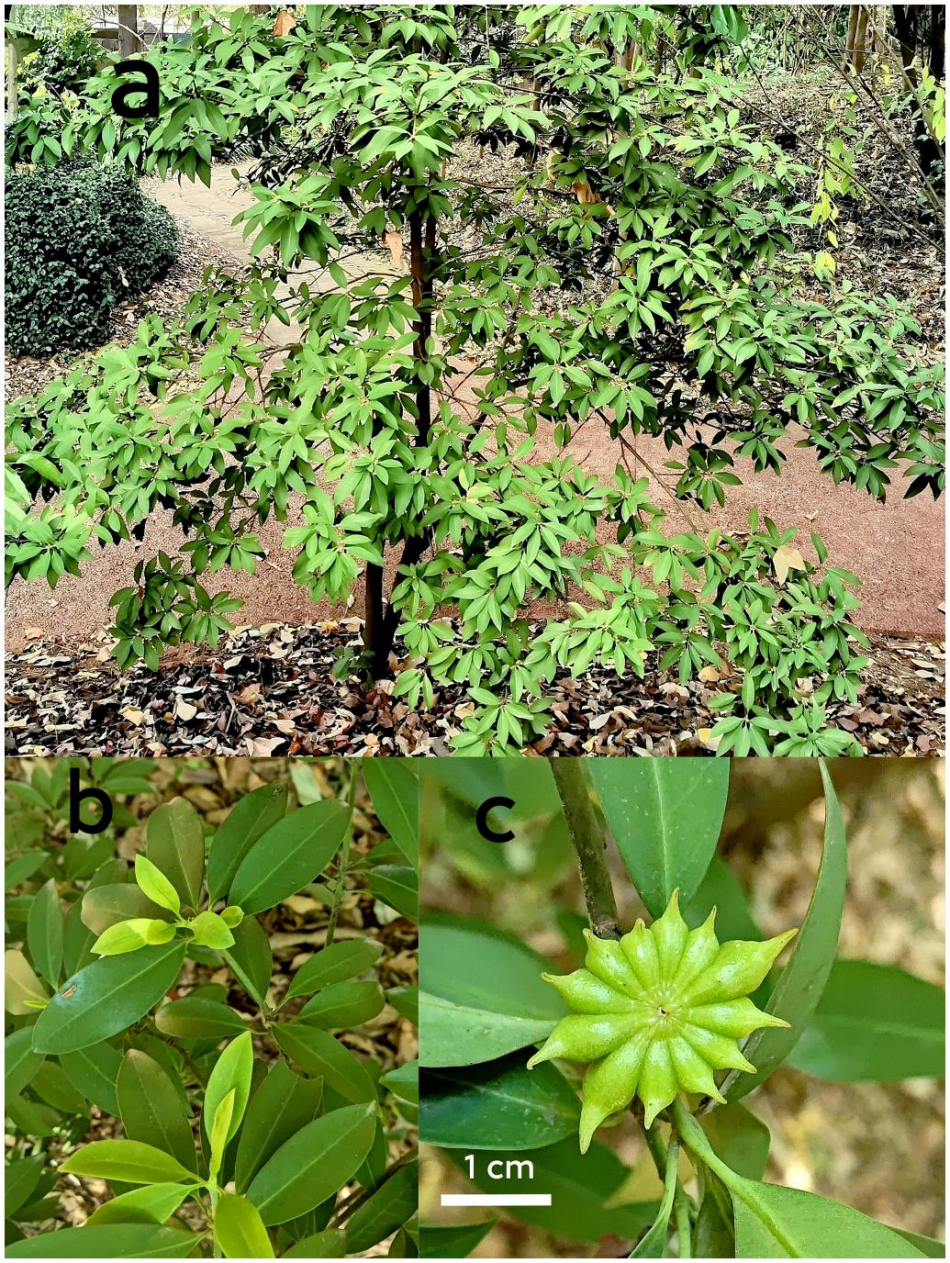
Images of the species *I. simonsii*. Images courtesy of corresponding author Yuan Huang. Fruit with 8–13 follicles. The leaves are leathery, lanceolate to ellipsoid, and lack stipules.

The sample of a single individual of *I. simonsii* was collected from Kunming Botanic Garden, Yunnan Province, China (102.71°E, 25.04°N; Figure 1). The voucher specimen was stored at the Herbarium of Yunnan Normal University (https://life.ynnu.edu.cn; Jianlin Hang, hjlynnu@163.com) under the voucher number y29.

**Figure 2.**
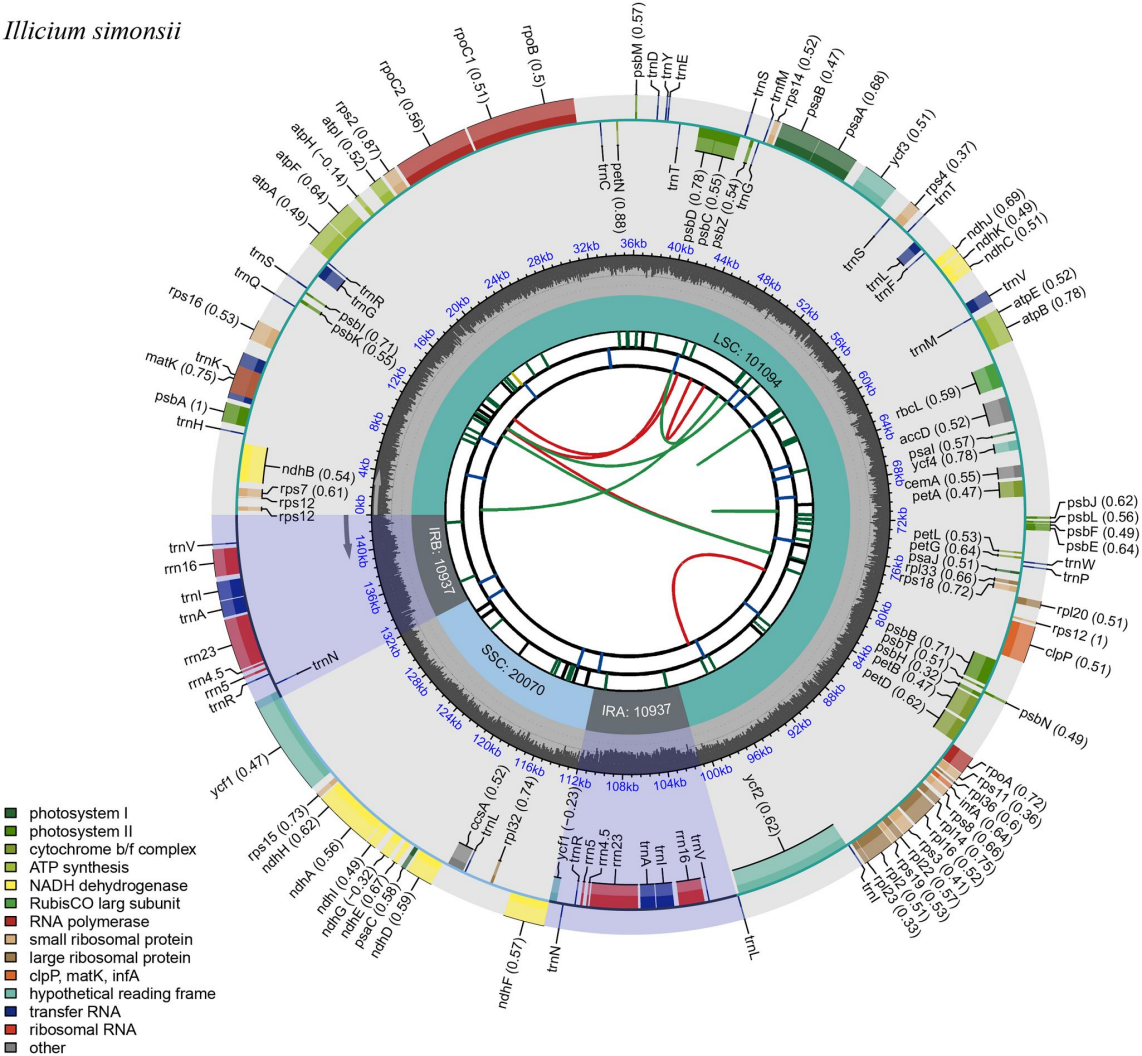
The complete chloroplast genome map of *I. simonsii.* This genome map was created using the CPGview. The species name was shown in the left top corner. The map contains six tracks by default. The GC content along the genome was plotted on the fifth track. The genes were shown on the sixth track. The optional codon usage bias was displayed in the parenthesis after the gene name. Genes were color-coded by their functional classification and were shown in the bottom left corner. The transcription directions for the inner and outer genes were clockwise and anticlockwise, respectively.

The total genomic DNA was extracted from the isolated chloroplasts using a modified CTAB method (Porebski et al. 1997) and stored in Huang’s laboratory at Yunnan Normal University. According to the criterion, we fragmented the DNA and used Illumina Hiseq X Ten sequencer to construct the genomic library for Illumina paired-end (PE) sequencing. We obtained about 3.8 Gb raw data and assembled the chloroplast genome of *I. simonsii* with NOVOPlasty v2.7.2 (Dierckxsens et al. 2016). Genious Primer v2023.0.1 was used to build a read coverage depth map to verify the accuracy of genome assembly (Figure S1). Then, the whole chloroplast genome sequence was corrected using BWA software (Wang et al. 2018). The complete chloroplast genome of *I. simonsii* was annotated using Geneious v2010.1.1 (Kearse et al. 2012), with *Illicium verum* as a reference genome (Genbank Accession No. KY085896). The genes or other feature-encoding regions were identified by BLAT-based homology searches, by profile HMM searches for protein and rRNA coding genes and two *de novo* predictors for tRNA genes. *Illicium henryi* (Genbank Accession No. KY085910) is utilized as a model for modification and improvement. The genome map (Figure 2) and gene structures (Figure S2) of its plastome were visualized using the CPGView online web (http://www.1kmpg.cn/cpgview; Liu et al. 2023). The boundaries of four regions of the complete chioroplast genome were compared using the online website IRscope (https://irscope.shinyapps.io/irapp/; Amiryousefi et al. 2018). DnaSP5.10 (Rozas et al. 2017) and mVISTA (Brudno et al. 2003) were used to compare the chloroplast genome sequences of 12 *Illicium* species (Table S1). To assess the divergence of the genome sequences, sliding windows with a window length of 600 bp and a step size of 200 bp were used to calculate nucleotide diversity.

The complete chloroplast genomes of 21 relative species were aligned using the program MAFFT v7.47 (Katoh and Standley 2013) to identify the phylogenetic position of *I. simonsii*. Then IQ_TREE v1.6.10 software was used to construct the maximum likelihood phylogenetic tree with 10,000 replicates (Nguyen et al. 2015). The best-fit model according to the Bayesian information criterion (BIC) is TIM + F+R2 (Kalyaanamoorthy et al. 2017).

## Results

**Figure 3.**
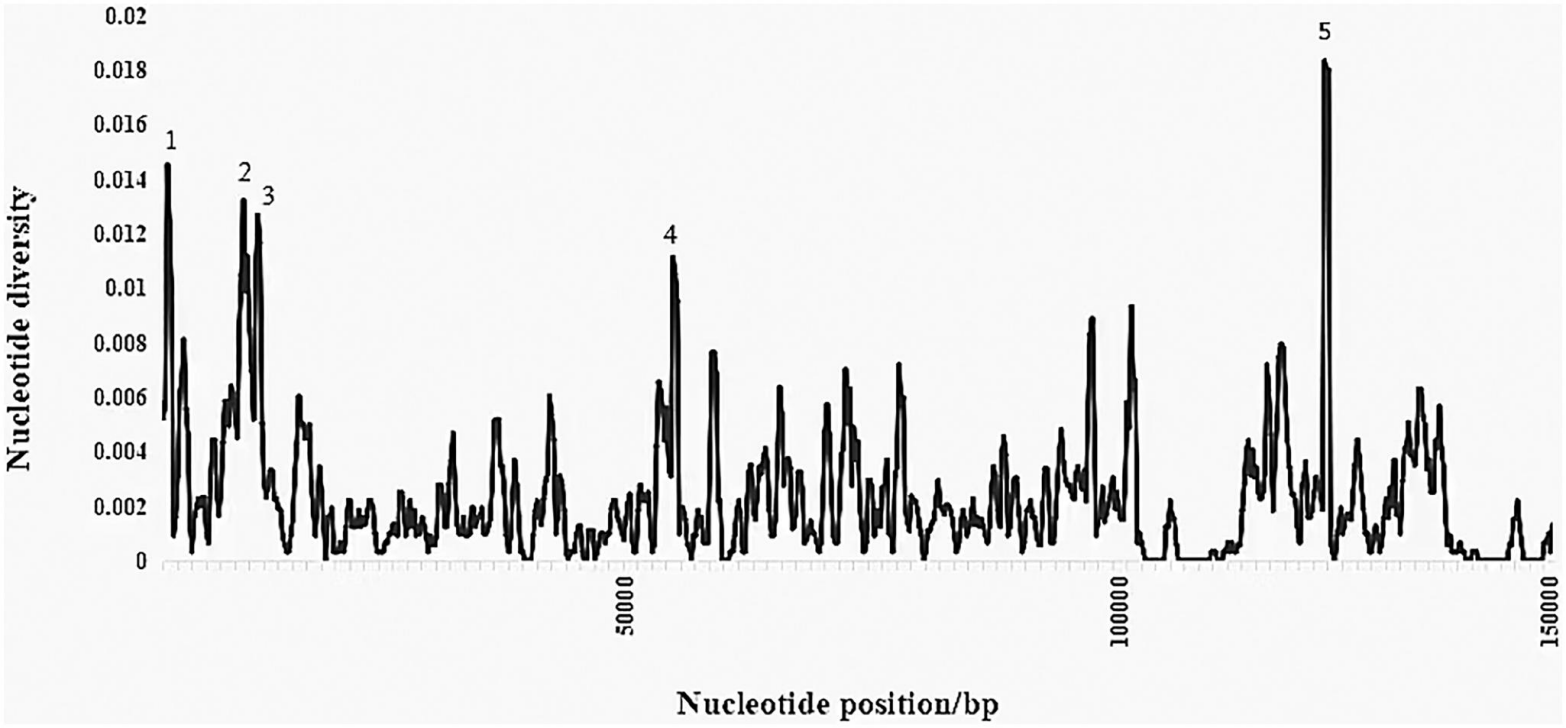
Results of sliding window analyses of 12 complete chloroplast genome sequences of the *illicium* species by using DnaSP 5.10. Five highly variation region were labeled individually with number.

The complete chloroplast genome of *I. simonsii* (GenBank Accession No. ON597624) was a circular DNA molecule with a length of 143,038 bp, containing 42,788 A bases (29.9%), 44,280 T bases (31.0%), 27,403 G bases (19.2%), 28,567 C bases (20.0%), respectively. The total GC content of the complete chloroplast genome was 39.1%. The genome contains a large single-copy (LSC) region of 101,094 bp, a small single-copy (SSC) region of 20,070 bp, and two inverted repeat (IR) regions of 21,874 bp (Figure 2). In total, the annotated chloroplast genome contains 113 genes, including 79 protein-coding genes, 30 tRNA genes, and four rRNA genes. Eleven genes, including *ndhB*, *rps16*, *atpF*, *rpoC1*, *ycf3*, *clpP*, *petB*, *petD*, *rpl16*, *rpl2* and *ndhA* were cis-splicing genes (Figure S2). Sliding window analysis using DnaSP 5.10 revealed five regions with higher nucleotide diversity than others (Figure 3). These regions include the intergenic spacer (IGS) regions of *rps16-trnQ* (1) and *ndhC-aptE* (4), as well as the protein coding regions of *aptA* (2), *aptF* (3) and *ycf1* (5). These 12 complete chloroplast genome sequences were visually analyzed using mVISTA (Figure S3), and the results were consistent with those of DnaSP.

**Figure 4.**
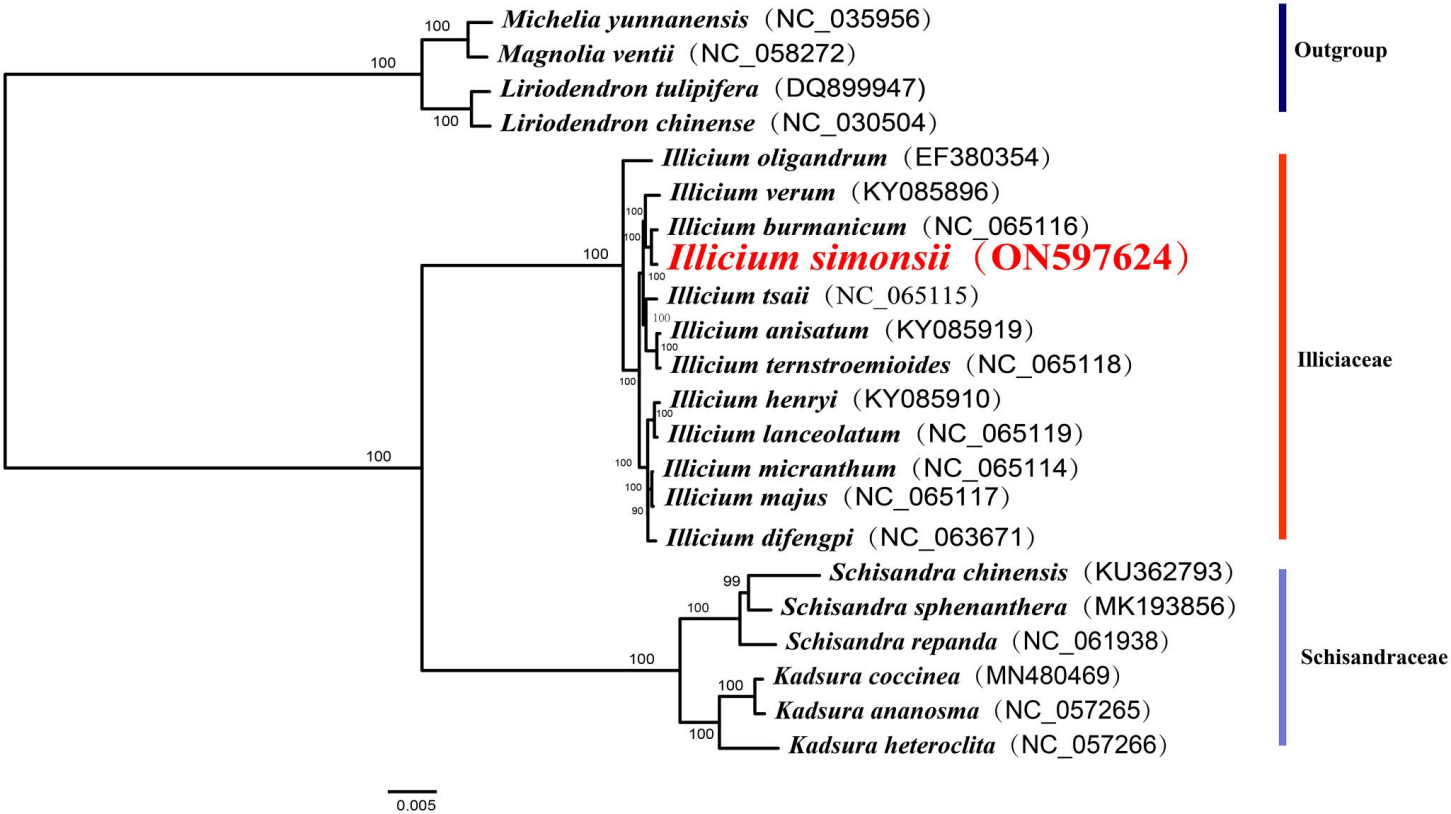
Maximum-likelihood phylogenetic tree of *I. simonsii* based on complete chloroplast genomes of 21 relative species, including *Liriodendron chinense* (NC_030504), *Liriodendron tulipifera* (DQ899947), *Magnolia ventii* (NC_058272), and *Michelia yunnanensis* (NC_035956), *I. oligandrum* (EF380354), *I. verum* (KY085896), *I. burmanicum* (NC_065116), *Illicium tsaii* (NC_065115), *Illicium anisntum* (KY085919), *Illicium ternstroemioides* (NC_065118), *Illicium henryi* (KY085910), *Illicium lanceolatum* (NC_065119), *Illicium micranthum* (NC_065114), *Illicium majus* (NC_065117), *Illicium difengpl* (NC_063671), *Schisandra chinensis* (KU362793), *Schisandra sphenanthera* (MK193856), *Schisandra repanda* (NC_061938), *Kadsura coccinea* (MN480469), *Kadsura ananosma* (NC_057265), *Kadsura heteroclita* (NC_057266). The name and accession number of species were listed behind each clade. The numbers above the branches indicate bootstrap support.

The phylogenetic tree (Figure 4) exhibited that *I. simonsii* was closely related to *I. burmanicum*, and formed a monophyletic clade with the other 10 species of the *Illicium* genus by 100% bootstrap value. Moreover, the species from the genus *Kadsura* and *Schisandra* formed monophyletic clades, respectively, were sister to the genus *Illicium.*

## Discussion and conclusion

In this study, the complete chloroplast genome of *I. simonsii* was sequenced, assembled and annotated. The total length is 14,308 bp, containing a large single-copy region (LSC) of 101,094 bp, a short single-copy region (SSC) of 20,070 bp, and a pair of inverted repeats (IRs) of 21,874 bp. The boundaries of the IR regions and the LSC and SSC regions of the complete chloroplast genomes of the 12 closely related species were compared (Figure S4), and found that fewer genes were observed to be duplicated in the genus *Illicium* due to the IR region contraction of more than 10 kb. This is consistent with the findings of a different study (Debra et al. 2007). Highly variable regions of the complete chloroplast genomes can be used to exploit markers to identify closely related species (Dong et al. 2014) and to provide abundant information for further phylogenetic study (Dong et al. 2012). In the genus *Illicium*, we have discovered five highly variable areas that could potentially serve as molecular markers in the future. This could facilitate more precise species identification and better resource usage of the genus *Illiccium*. The phylogenetic tree constructed based on the complete chloroplast genome showed that species in the genus *Illicium* formed a monophyly and were closely related to the Schisandraceae which include the genus *Schisandra* and *Kadsura*. This conclusion is in line with earlier phylogenetic research (Hu 1950; Li and Zheng 2018).

In summary, the complete chloroplast genome of *I. simonsii* could provide a valuable genomic resource for species identification and further phylogenetic studies of *Illicium* species.

## Supplementary Material

Supplemental Material

Supplemental Material

Supplemental Material

Supplemental Material

## Data Availability

The genome sequence data supporting this study’s findings are openly available in GenBank of NCBI at [https://www.ncbi.nlm.nih.gov/] under accession no. ON597624. The associated BioProject, SRA, and Bio-Sample numbers are PRJNA841840, SRR19392935, and SAMN28626151, respectively.
