# Supplementary figures and images for: The complete chloroplast genome of Illicium simonsii Maxim. (Illiciaceae), a species with important medicinal properties
Source: Mitochondrial DNA B Resour. 2024 May 22;9(5):678–82. doi: 10.1080/23802359.2024.2356753 (PMC11123442; doi:10.1080/23802359.2024.2356753)

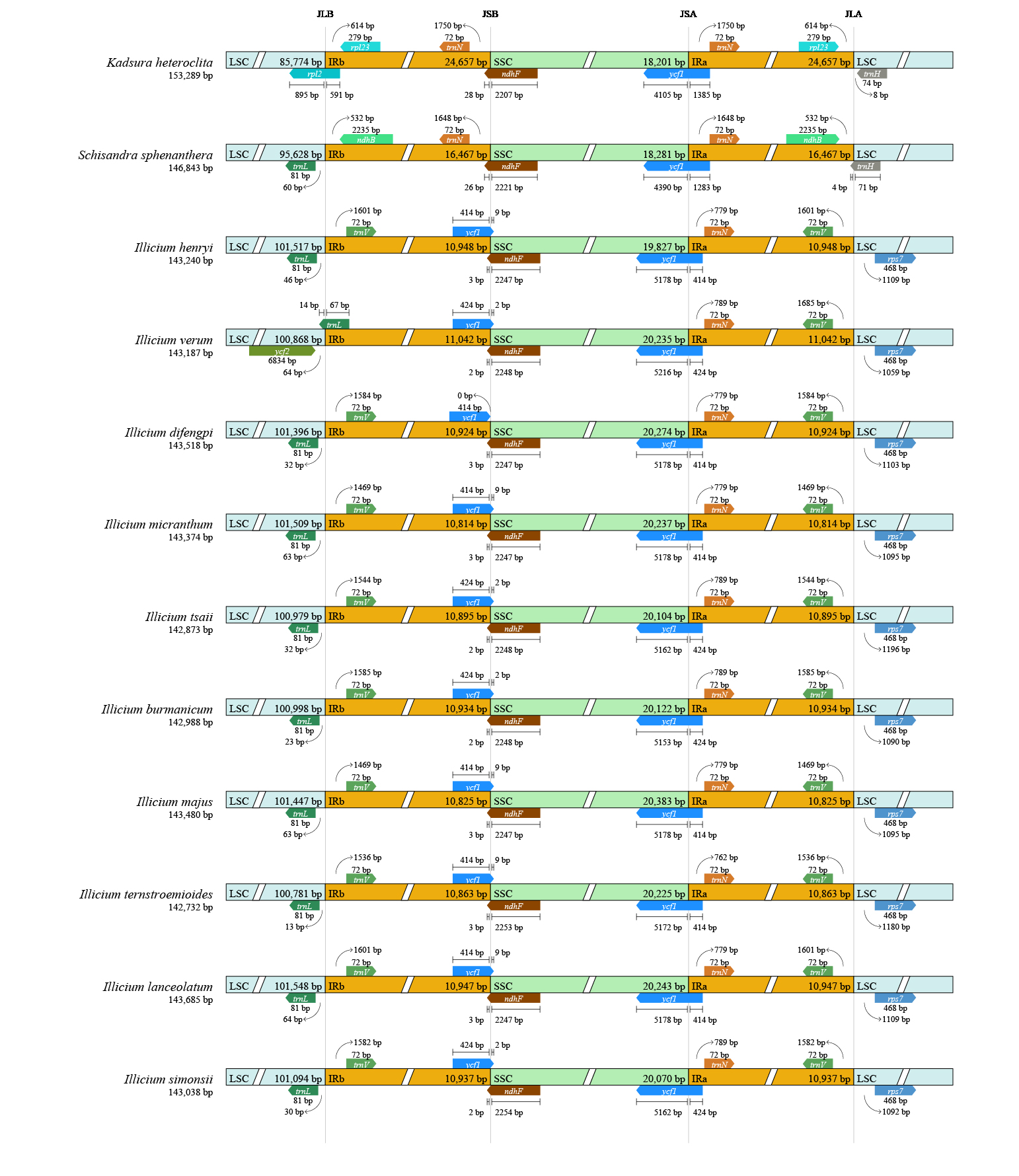

Supplement: Supplemental Material [file TMDN_A_2356753_SM8988.jpg]

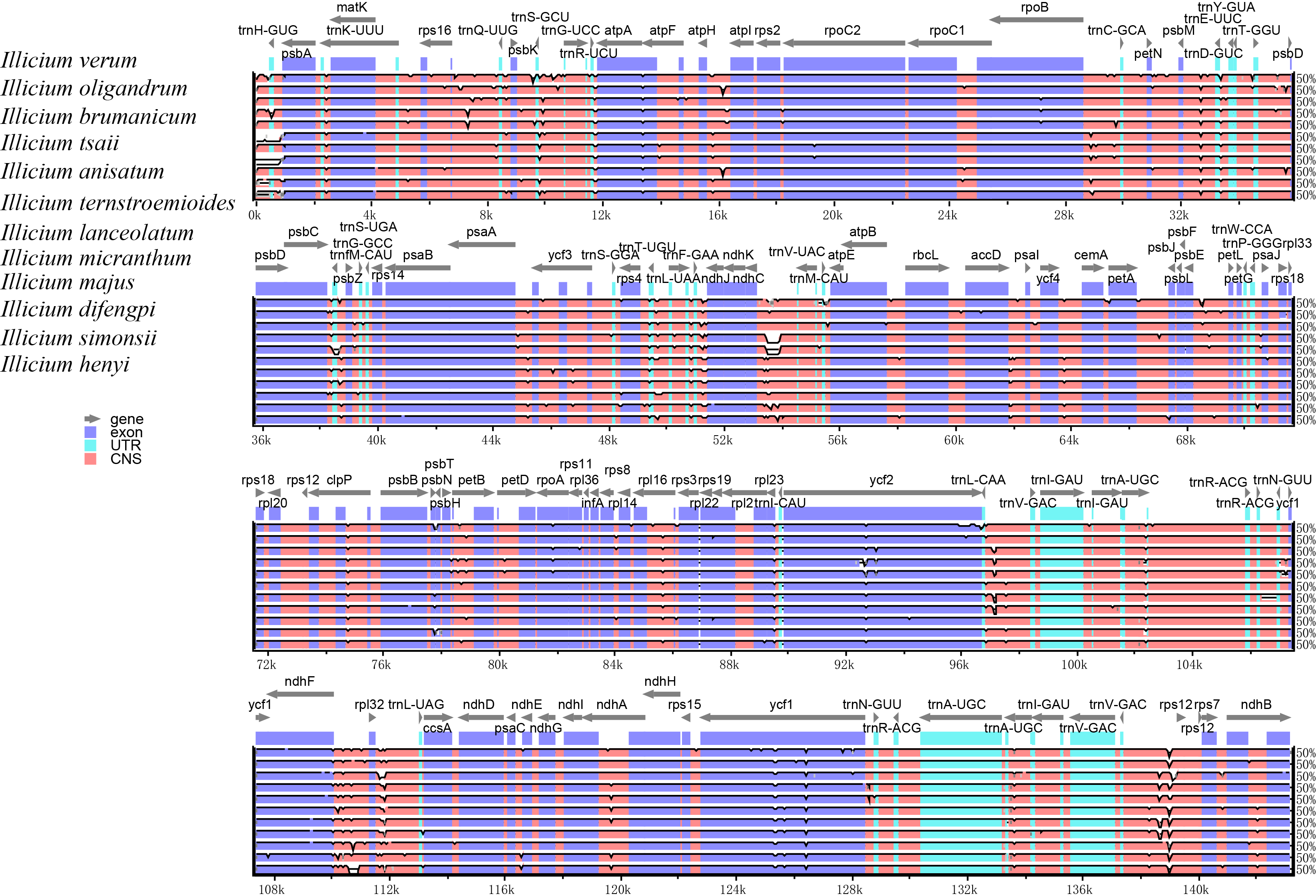

Supplement: Supplemental Material [file TMDN_A_2356753_SM8985.png]

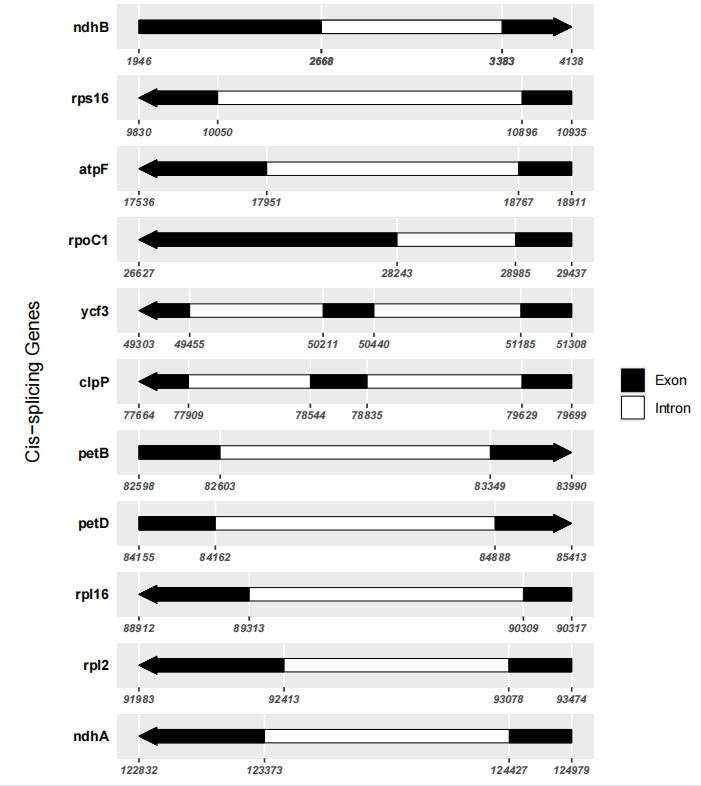

Supplement: Supplemental Material [file TMDN_A_2356753_SM8984.png]

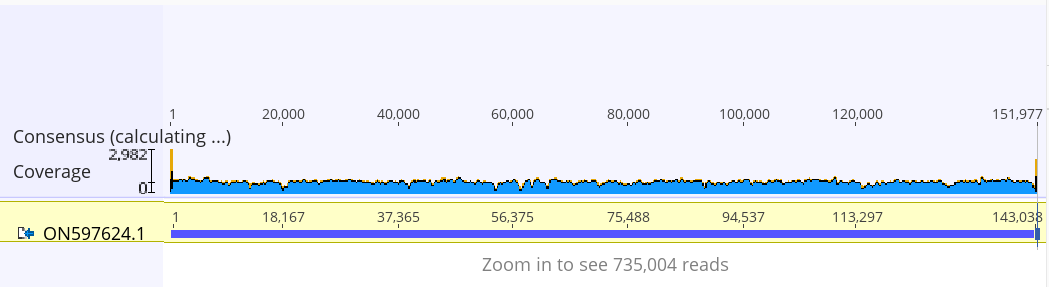

Supplement: Supplemental Material [file TMDN_A_2356753_SM8982.png]
